# Editorial: Tumor microenvironment in primary brain cancers

**DOI:** 10.3389/fonc.2023.1136895

**Published:** 2023-01-24

**Authors:** Ignacio Niechi, Jose R. Pineda

**Affiliations:** ^1^ Tumor biology laboratory, Institute of Biochemistry and Microbiology, Faculty of Sciences, Universidad Austral de Chile, Valdivia, Chile; ^2^ Millennium Institute on Immunology and Immunotherapy, Universidad Austral de Chile, Valdivia, Chile; ^3^ Signaling Lab, Department of Cell Biology and Histology, Faculty of Medicine and Nursing, University of the Basque Country (UPV/EHU), Leioa, Spain; ^4^ Achucarro Basque Center for Neuroscience Fundazioa, Leioa, Spain

**Keywords:** Tumor microenvironment, brain cancer, tumor malignancy, extracellular matrix, CD44, brain parenchyma

Primary brain tumors are most commonly detected in the late stages of the disease and there are only a few treatment opportunities. Overall survival rates are significantly low and the response to chemo/radiotherapy is not sufficient, despite surgical intervention. The tumor microenvironment (TME) regulates several brain tumor hallmarks, such as cell migration, invasiveness, proliferation, therapy resistance, stemness maintenance, immune evasion, among others. Therefore, it is necessary to understand the pro-tumoral mechanisms that are regulated by the TME in order to detect and identify new biomarkers and novel therapeutic targets.

An aim of the Research Topic “*Tumor Microenvironment in Primary Brain Cancers*” is to generate a discussion regarding the most recent advances in the interaction of the TME with primary brain tumors. The understanding of the key aspects in the interaction of the TME with cancer cells is important in the search for new markers to improve early detection, survival prognostic and therapy prediction. In this Research Topic we reported that Serum Amyloid A1 (SAA1) may serve as a biomarker to predict prognosis and to classify TME status between different subtype of gliomas. SAA1 participates in cell cycle and mitosis; however, the described changes in its levels of expression provokes a gain of function to regulate immune activity. Indeed, its overexpression upregulated, in turn, LAIR1 and TNFSF14 affecting response in turn to glioblastoma immune therapy (Cao et al.). This Research Topic also combined approaches of deep genome sequencing and tumor transcriptome crossed with the clinical assessment of patient cohorts to identify new biomarkers and susceptible therapeutic targets for personalized potential treatments. In this line and using unbiased *in silico* data-minig strategy it has been found that the signaling hub *PREX1* signature has a strong correlation with the prognosis of low grade glioma patients and could be a new therapeutic approach especially relevant for immunotherapeutic strategies (Beltrán-Navarro et al.). One of the main hallmarks of poor prognosis and high rate of GBM recurrence is the high capacity of GBM cells to extensively infiltrate the surrounding brain parenchyma. This results in a diffuse spreading of neoplastic tumor cells to distant parts of the brain. One of the key proteins overexpressed in glioma as well as multiple cancer types is the CD44 (Cluster of Differentiation 44) transmembrane protein implicated in cell proliferation, angiogenesis and cell motility including motility through the vasculature. To date, several studies have been characterized the role of CD44 in glioblastoma. Despite a clear correlation between CD44 expression levels and tumor prognosis that established a clear direct positive correlation, the role of CD44 on TME is currently unknown. In our Research Topic, the potential role of CD44 in the TME was explored using *ex vivo* invasion assays of organotypic brain slice co-cultured with tumor cells. Complementary to these approaches it has been put into evidence that CD44 expression in TME also modulates tumor phenotype. Genetic ablation of CD44 expressed in TME results in an impairment of the invasive capacity of glioma cells. On the other hand, a genetic ablation of CD44 in different cell types such as astrocytes and endothelial cells does not provoke the same phenotype that genetic ablation in myeloid cells and primary microglia. Indeed, in these cells CD44 invasive properties signals through Toll Like Receptor 2 signaling becoming a major regulator of Matrix Metalloproteinase 9 (MMP9) expression (Ivanova et al.). Other membrane bound molecules able to enhance the invasive, migratory and angiogenic capacity of glioblastoma stem-like cells (GSCs) are the low affinity A_2B_ adenosine receptor (A_2B_AR). GSCs are a subpopulation of cells whose niche resides in hypoxic regions. GSCs have high capacity to infiltrate into brain parenchyma and are responsible for tumor recurrence. The hypoxic microenvironment is able to increase extracellular adenosine levels, activating the low affinity A_2B_AR and correlating with high-grade glioma and hypoxic/necrotic areas. The strategy to block A_2B_AR using specific antagonists to decrease both VEGF expression and blood vessel formation highlights the possibility to be used as a therapeutic target for GBM recurrence (Erices et al.).

Finally, to sum up the interactions of the microenvironment, including the non-cellular components of brain tumors, and the tumoral cellular components we added a systematic review showing the microenvironmental landscape and its therapeutic implications. Briefly, the supportive tissue of the peripheral nervous system stroma contains extracellular matrix (ECM) such as fibrillary collagens, laminins, and fibronectin. In a healthy environment, it is also composed of a balanced proportion of proteo-, glycosamino- glycans and glycoproteins and fibrillary components associated with the vasculature. However, in recent studies that characterized the composition of TME in GBM, there is an unbalance, and shift of ECM component proportions towards upregulated collagen synthesis and aligned fibrillary ECM networks. One of the most well-known interactions between tumor cells and the microenvironment is the capacity of tumor cells to degrade the ECM and brain parenchyma to allow either individual or collective cell invasion to the adjacent regions of brain tissue. Both ECM degradation and cell-to-cell communication with other tumor cells or the microenvironment is driven through physical and chemical control of their environment. To achieve that, tumor cells rely on the modulation of cell signaling cascades either in normal or poisoned microenvironment such as that produced by chemo- and radiotherapies for their own benefit. As a consequence, tumor cells acquire radio- and chemo-resistance allowing tumor relapse and subsequent cancer progression. Gaining knowledge of these interactions and targeting key components specifically upregulated in cancer cells is an attractive strategy for overcoming therapy resistance and has been focused in an interesting review of Faisal et al. culminating the Research Topic “ Tumor Microenvironment in Primary Brain Cancers”.

## Author contributions

All authors listed have made a substantial, direct, and intellectual contribution to the work and approved it for publication.

